# Is locomotion training effective for middle‐aged workers?

**DOI:** 10.1002/1348-9585.12303

**Published:** 2021-12-20

**Authors:** Akinobu Nishimura, Makoto Ohtsuki, Toshihiro Kato, Rie Nagao‐Nishiwaki, Yoshiyuki Senga, Ko Kato, Toru Ogura, Akihiro Sudo

**Affiliations:** ^1^ Department of Orthopaedic Surgery Mie University Graduate School of Medicine Tsu City, Mie Japan; ^2^ Department of Orthopaedic and Sports Medicine Mie University Graduate School of Medicine Tsu City, Mie Japan; ^3^ Department of Clinical Nutrition Suzuka University of Medical Science Suzuka City, Mie Japan; ^4^ Department of Rehabilitation Suzuka Kaisei Hospital Suzuka City, Mie Japan; ^5^ Department of Nursing Faculty of Health Science Suzuka University of Medical Science Suzuka City, Mie Japan; ^6^ Department of Orthopaedic Surgery Suzuka Kaisei Hospital Suzuka City, Mie Japan; ^7^ Department of Clinical Research Support Center Mie University Graduate School of Medicine Tsu City, Mie Japan

**Keywords:** epidemiology, locomotion training, locomotive syndrome, young and middle adulthood

## Abstract

**Objectives:**

Locomotion training (LT) consisting of single‐leg standing and squatting was developed to help prevent locomotive syndrome (LS), and is typically used in older people. The objective of this study was to examine the effects of LT on young and middle‐aged people.

**Methods:**

This study was performed at two companies. Workers in company A engaged in LT five times/week for 1 year, whereas workers in company B did not. Baseline and follow‐up checkups consisted of questionnaires and physical performance tests, including three kinds of locomotion tests.

**Results:**

In total, 88 and 101 workers in companies A and B, respectively, met the inclusion criteria. LS stage, stand‐up test results, and scores on a geriatric locomotive function scale significantly improved among workers in company A, but only stand‐up test results significantly improved among workers in company B. Quadriceps power increased in company A, but did not change in company B. Especially, workers with LS in company A had more significant changes than those without LS and those in company B.

**Conclusions:**

The results of this longitudinal study suggest that LT is useful even for young and middle‐aged workers. LT was especially more effective for workers than those without LS.

## 1 INTRODUCTION

The older population (age 65 years or older) in Japan has rapidly increased, exceeding 21% in 2007, making it the earliest super‐aged society.^1^ This rate is expected to reach 38.4% by 2065. The aging of the population has led to the increased prevalence of various diseases, and in turn, increasing costs for nursing care. Motor organ problems are one of the most important causes of requiring nursing care. Falls and fractures, joint diseases, and spinal cord injuries have been reported to account for 12.5%, 10.2%, and 2.3% of all causes for requiring nursing care in Japan.^2^ The Japanese Orthopaedic Association (JOA) has advocated the term “locomotive syndrome (LS)” to define a state of degraded mobility due to impaired locomotive organs and an elevated risk of disability.^3^ LS is assessed by three simple tests: a questionnaire (the 25‐question Geriatric Locomotive Function Scale [GLFS‐25]), a two‐step test, and a stand‐up test.^3^ The symptoms of LS can worsen the quality of life and impair activities of daily living.^4^ In addition, older people with LS are at an increased risk of falling.^5^ The JOA has also advocated locomotion training (LT), which consists of squatting and single‐leg standing with eyes open to prevent LS. LT exercises are recommended because they are directly related to standing and gait function^6^ and are safe and feasible to perform at home for self‐management. LT has also been shown to be effective for preventing or improving LS in older people.^7^, ^8^ Originally, these LT exercises were developed for older people, as few cases of LS in young and middle‐aged adults have been reported.^9^ A previous study^9^ found that about 20% of young and middle‐aged workers had LS. From this point of view, the early detection of, and early interventions for, LS are important for decreasing the number of older people who will require nursing care in the future. However, to our knowledge, no studies have investigated whether LT is effective for young and middle‐aged people. Therefore, the purpose of this epidemiological study was to examine the effects of LT on young and middle‐aged people, including workers age between 30 and 64 years.

Exercise habits represent another important factor in preventing LS, as LS occurs less frequently in people than in those without good exercise habits.^10^, ^11^ Therefore, another purpose of this study was to examine whether LT improves exercise habits among workers.

## 2 MATERIALS AND METHODS

This study was performed at two companies recruited by the public health department of a local prefecture in Japan that agreed to participate. Company A was a drug company and Company B was a chemical company. The health departments of these two companies recruited employees to join health examinations and provided some rooms for the checkups. In total, 88 employees in company A and 101 employees in company B underwent the checkups. All participants provided written informed consent prior to participating. This study was approved by the Institutional Review Board of the authors’ affiliated institutions.

The checkups in this study consisted of questionnaires and physical performance tests. The questionnaires included items on age, sex, lifestyle, physical activity, changes in exercise behavior, and the GLFS‐25. Changes in exercise behavior were assessed using the following question from Oka's questionnaire^12^: “Could you tell me your exercise habits (a bout of exercise is considered exercising for more than 20 minutes at a time more than twice/week)?” The five responses that can be given to this question are as follows:

1. “I do not exercise now and I have no plan to exercise in the future.”
2. “I do not exercise now but I have a plan to start to exercise in the near future (in 6 months).”
3. “I exercise now but not regularly (less than twice/week).”
4. “I exercise regularly now (at least twice/week). However, I have been doing this for less than 6 months.”
5. “I exercise regularly now (at least twice/week), and I have been doing this for more than 6 months.”

These choices correspond to different stages of exercise behavior, with choices 1 through 5 representing the precontemplation, contemplation, preparation, action, and maintenance stages, respectively. To evaluate changes in exercise behavior, the responses were scored from 1 to 5 according to the level of exercise activity (in order from precontemplation = 1 to maintenance = 5). The GLFS‐25 is a self‐administered questionnaire developed by Seichi et al.^13^ to evaluate LS. This questionnaire is composed of 25 questions, including four on pain during the last month, 16 on activities of daily activity living during the last month, three on social functions, and two on mental health status during the last month. These 25 questions are graded on a five‐point scale, from no impairment (0 points) to severe impairment (4 points), for a total possible score of 100 (with 0 being the healthiest and 100 the least healthy).

Physical tests consisted of those for quadriceps muscle strength, a two‐step test, and a stand‐up test. Quadriceps muscle strength was evaluated using the Locomo Scan rehabilitation device (ALCARE Co. Ltd). According to the instructions in a previous report,^14^ after receiving an explanation regarding how to apply the device to a participant's quadriceps muscle, the tester measured the participant's maximum quadriceps muscle strength for 10 s twice for each leg, and recorded the better value of the two measurements.

In the two‐step test, participants move forward with the widest length of two strides from a starting line while being careful not to lose their balance. The two‐step test score is then calculated by dividing the distance by the participant's height. The test was performed twice, and the best result was recorded.

The stand‐up test assesses lower muscle strength by having the participant stand on one or both legs from a seat with a specified height (40, 30, 20, and 10 cm in order of difficulty from easy to difficult for the double‐leg stance followed by the single‐leg stance). The result is reported as the minimum height from which a participant can stand up. To evaluate the stand‐up test quantitatively, the results were scored from 0 to 8 according to the level of difficulty (e.g., fail at 40 cm with the double‐leg stance = 0, stand from 40 cm with the double‐leg stance = 1, stand from 10 cm with the single‐leg stance = 8), as described in Ogata's report.^15^

According to the results of the three assessments (the GLFS‐25, the two‐step test, and the stand‐up test), the LS risk level was determined as follows^16^:

Stage 1 (LS‐1):

1. Two‐step test score <1.3.
2. Difficulty standing from a 40‐cm‐high seat using one leg (either leg) in the stand‐up test.
3. GLFS‐25 ≥7.

When a participant met any of the above‐mentioned criteria, he/she was diagnosed as LS stage 1.

Stage 2 (LS‐2):

1. Two‐step test score <1.1.
2. Difficulty standing from a 20‐cm‐high seat using both legs in the stand‐up test.
3. GLFS‐25 ≥16.

When a participant met any of the above‐mentioned criteria, he/she was diagnosed as LS stage 2.

Stage 3 (LS‐3):

1. Two‐step test score <0.9.
2. Difficulty standing from a 30‐cm‐high seat using both legs in the stand‐up test.
3. GLFS‐25 ≥24.

**FIGURE 1:**
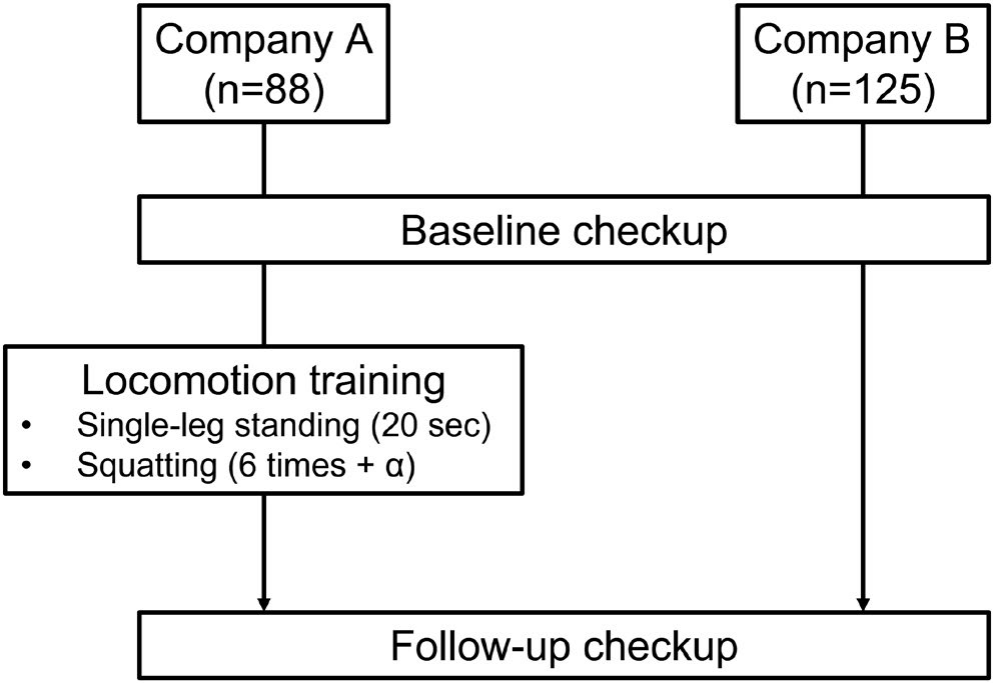
Flowchart of the study design and participant selection

After the baseline checkup, workers in company A started LT, which was held in the morning assembly (Figure 1). The JOA originally recommended performing one‐leg standing with the eyes open for 60 s on each leg and three sets of squats (six repetitions) per day.^3^ However, company A wanted the whole LT program to take only about 1 min because they did not want it to interfere with their working time. Therefore, we modified the LT to consist of single‐leg standing with the eyes closed for 20 s on each leg and at least six repetitions of squats. We gave a lecture about LS and LT to the workers in company A before the intervention and advised them to perform more than six repetitions of squats until they felt muscle fatigue. For company B, we also gave a lecture about LS and LT after the baseline checkup. The workers usually worked 5 days/week; therefore, they engaged in LT five times/week. The LT intervention continued for 12 months, after which, follow‐up exams were given.

### 2.1 Statistical analysis

Means and standard deviations are summarized for all continuous variables unless otherwise noted. All data were statistically analyzed using PASW Statistics for Windows, version 25 (SPSS Inc., IBM Corp.). Differences in continuous and categorical variables between Companies A and B were evaluated using an unpaired *t*‐test and the chi‐squared test, respectively. Differences in continuous values such as quadriceps muscle strength, the two‐step test, and the GLFS‐25 between baseline and follow‐up in both groups were tested for significance using a paired *t*‐test. Differences in ordinal variables such as the stand‐up test and changes in exercise behavior between baseline and follow‐up were tested using the Wilcoxon signed‐rank test. LS stage between baseline and follow‐up were tested using Fisher's exact test. The interaction between time and group for scores on the two‐step test, stand‐up test, GLFS‐25, and muscle strength was analyzed by repeated‐measures ANOVA. Significance was determined at a level of 5% for all tests.

## 3 RESULTS

Table 1 shows the physical characteristics of the participants in both groups. No significant differences were found between groups in sex, age, height, weight, or BMI. The implementation rate of LT (i.e., the percentage of workers coming to work in the morning) was 80.3%.

**TABLE 1:** Physical characteristics of each group

	Company A, *n* = 88	Company B, *n* = 101	*P* value
Sex (male/female)	71/17	76/25	.387
Age (years)	46.0 ± 7.4	46.9 ± 8.8	.418
Height (cm)	168.0 ± 8.8	167.1 ± 8.2	.460
Weight (kg)	65.3 ± 11.3	64.3 ± 9.7	.511
BMI (kg/m^2^)	23.0 ± 2.6	23.0 ± 2.6	.938

Note

Data are expressed as the mean ± standard deviation for age, height, weight, and BMI. Differences in age, height, weight, and BMI between the two groups were analyzed for significance using an unpaired *t*‐test, and differences in sex were analyzed using Pearson's chi‐squared test.

Abbreviations: BMI, body mass index; *n*, number.

Table 2 shows the results of the three locomotion tests, LS stage, and muscle strength for both groups. LS stage was significantly improved in Company A (baseline: stage 0/1/2/3 = 67/20/1/0; follow‐up: stage 0/1/2/3 = 80/6/2/0; *P* = .022), but no significant changes were seen in Company B (baseline: stage 0/1/2/3 = 88/11/2/0; follow‐up: stage 0/1/2/3 = 84/15/2/0; *P* = .320). No significant changes were seen in the two‐step test scores for either group, but significant improvement was seen in the stand‐up test for both groups. GLFS‐25 scores (mean ± standard deviation [SD]) significantly improved for Company A (baseline: 3.88 ± 3.54; follow‐up: 2.72 ± 2.81; *P* = .002), but not for Company B (baseline: 3.64 ± 5.30; follow‐up: 3.28 ± 3.84; *P* = .325). The baseline and follow‐up values for quadriceps muscle strength were 501.8 ± 132.5 N and 525.4 ± 126.4 N, respectively, for Company A, and 526.7 ± 128.7 N and 518.0 ± 134.0 N, respectively, for Company B. No significant changes were found between baseline and follow‐up scores for Company B, but a significant increase was seen for Company A (*P* = .010).

**TABLE 2:** Comparison of locomotive syndrome (LS) stage and LS tests between baseline and follow‐up for both companies

	Company A (*n* = 88)			Company B (*n* = 101)		
	Baseline	Follow‐up	*P* value	Baseline	Follow‐up	*P* value
LS stage						
Stage 0/1/2/3	67/20/1/0	80/6/2/0		88/11/2/0	84/15/2/0	
Median (IQR)	0 (0–0)	0 (0–0)	.022*	0 (0–0)	0 (0–0)	.32
Two‐step test score						
Mean ± SD	1.66 ± 0.12	1.66 ± 0.11	.822	1.65 ± 0.12	1.66 ± 0.12	.064
Stand‐up test						
Median (IQR)	6 (5–8)	6 (5–8)	.012*	6 (5–7)	6 (5–8)	.042*
GLFS‐25						
Mean ± SD	3.88 ± 3.54	2.72 ± 2.81	.002*	3.64 ± 5.30	3.28 ± 3.84	.325
Pain						
Mean ± SD	1.97 ± 2.21	1.59 ± 1.62	.070	1.68 ± 1.90	1.63 ± 1.93	.741
Activities						
Mean ± SD	0.63 ± 1.11	0.42 ± 1.36	.016*	0.78 ± 2.50	0.57 ± 1.57	.384
Social functions						
Mean ± SD	1.09 ± 1.82	0.58 ± 1.21	.003*	0.86 ± 1.47	0.80 ± 1.38	.716
Mental health						
Mean ± SD	0.25 ± 0.58	0.13 ± 0.40	.052	0.32 ± 0.87	0.27 ± 0.71	.334
Muscle strength (N)						
Mean ± SD	501.8 ± 132.5	525.4 ± 126.4	.010*	526.7 ± 128.7	518.0 ± 134.0	.209

Abbreviations: GLFS‐25, 25‐question Geriatric Locomotive Function Scale; IQR, interquartile range; LS, locomotive syndrome; SD, standard deviation.

* *P* < .01.

The numbers of participants with LS at baseline were 21 in Company A and 13 in Company B. Table 3 shows the results regarding LS stage, the three locomotion tests, and quadriceps muscle strength at baseline and follow‐up among the participants with LS for both companies. LS stage (baseline: stage 0/1/2/3 = 0/20/1/0; follow‐up: stage 0/1/2/3 = 16/5/0/0; *P* < .001) and GLFS‐25 scores (baseline: 8.38 ± 3.75; follow‐up: 4.67 ± 2.97; *P* = .001) significantly improved among the participants with LS in Company A; however, no significant differences were seen in Company B.

**TABLE 3:** Comparison of locomotive syndrome (LS) stage and LS tests between baseline and follow‐up for workers with LS for both companies

	LS in Company A (*n* = 21)			LS in Company B (*n* = 13)		
	Baseline	Follow‐up	*P* value	Baseline	Follow‐up	*P* value
LS stage						
Stage 0/1/2/3	0/20/1/0	16/5/0/0		0/11/2/0	5/6/2/0	
Median (IQR)	1 (1–1)	0 (0–0.5)		1 (1–1)	1 (0–1)	
Two‐step test score						
Mean ± SD	1.63 ± 0.13	1.61 ± 0.10	.210	1.63 ± 0.13	1.65 ± 0.12	.228
Stand‐up test						
Median (IQR)	5 (4–7.5)	5 (5–8)	.059	5 (5–7)	6 (5–8)	.683
GLFS‐25						
Mean ± SD	8.38 ± 3.75	4.67 ± 2.97	.001*	13.15 ± 9.92	9.31 ± 6.16	.126
Pain						
Mean ± SD	4.14 ± 2.87	2.33 ± 1.49	.002*	4.62 ± 2.18	4.08 ± 2.78	.552
Activities						
Mean ± SD	1.57 ± 1.63	1.00 ± 1.87	.096	4.31 ± 5.88	2.38 ± 3.52	.12
Social functions						
Mean ± SD	2.24 ± 2.36	1.00 ± 1.58	.008*	2.77 ± 2.62	1.54 ± 1.98	.041*
Mental health						
Mean ± SD	0.43 ± 0.81	0.33 ± 0.66	.589	1.46 ± 1.85	1.31 ± 1.38	.680
Muscle strength (N)						
Mean ± SD	458.1 ± 105.6	482.3 ± 131.6	.298	501.2 ± 125.0	506.7 ± 184.7	.819

Abbreviations: GLFS‐25, 25‐question Geriatric Locomotive Function Scale; IQR, interquartile range; LS, locomotive syndrome; SD, standard deviation.

* *P* < .01.

The numbers of participants without LS at baseline were 67 in Company A and 88 in Company B. Table 4 shows results regarding LS stage, the three locomotion tests, and quadriceps muscle strength at baseline and follow‐up among the participants without LS for both companies. In Company A, muscle strength significantly increased and performance in the stand‐up test tended to be improved at follow‐up compared with baseline (*P* = .065). On the other hand, for Company B, LS stage at follow‐up was significantly worse than that at baseline (baseline: stage 0/1/2 = 88/0/0/0; follow‐up: stage 0/1/2/0 = 79/9/0; *P* = .003). However, the results of the stand‐up test for Company B were significantly improved at follow‐up compared with baseline (*P* = .031). Regarding changes in the stand‐up test, 22 were improved, 55 were unchanged, and 11 were worse. Most of the participants who showed improvement on the stand‐up test were LS stage 0 in the baseline survey; fewer participants who were LS stage 1 or 2 in the baseline survey showed improvement on the stand‐up test.

**TABLE 4:** Comparison of locomotive syndrome (LS) stage and LS tests between baseline and follow‐up for workers without LS for both companies

	No LS in Company A (*n* = 67)			No LS in Company B (*n* = 88)		
	Baseline	Follow‐up	*P* value	Baseline	Follow‐up	*P* value
LS stage						
Stage 0/1/2/3	67/0/0/0	64/1/2/0		88/0/0/0	79/9/0/0	
Median (IQR)	0 (0–0)	0 (0–0)	.102	0 (0–0)	0 (0–0)	.003*
Two‐step test score						
Mean ± SD	1.67 ± 0.12	1.67 ± 0.11	.395	1.65 ± 0.12	1.67 ± 0.12	.106
Stand‐up test						
Median (IQR)	6 (5–8)	6 (5–8)	.065	6 (5–7)	6 (5–8)	.031**
GLFS‐25						
Mean ± SD	2.46 ± 1.91	2.10 ± 2.48	.277	2.24 ± 1.81	2.39 ± 2.34	.492
Pain						
Mean ± SD	1.28 ± 1.40	1.36 ± 1.59	.929	1.25 ± 1.42	1.27 ± 1.48	.950
Activities						
Mean ± SD	0.33 ± 0.66	0.24 ± 1.14	.077	0.26 ± 0.56	0.31 ± 0.75	.634
Social functions						
Mean ± SD	0.73 ± 1.45	0.45 ± 1.05	.092	0.58 ± 0.96	0.69 ± 1.24	.498
Mental health						
Mean ± SD	0.19 ± 0.47	0.06 ± 0.24	.039*	0.15 ± 0.42	0.11 ± 0.35	.257
Muscle strength (N)						
Mean ± SD	515.5 ± 137.8	539.0 ± 122.6	.016*	530.4 ± 129.5	519.6 ± 126.2	.133

Abbreviations: GLFS‐25, 25‐question Geriatric Locomotive Function Scale; IQR, interquartile range; LS, locomotive syndrome; SD, standard deviation.

* *P* < .01.

** *P* < .05.

The baseline and follow‐up values (mean ± SD) for exercise behavior in Company A were 2.83 ± 1.38 and 2.80 ± 1.32, respectively, while those in Company B were 2.83 ± 1.41 and 2.66 ± 1.40, respectively. No significant differences were seen between the baseline and follow‐up scores in either group.

## 4 DISCUSSION

In this study, we investigated the effects of LT for middle‐aged workers. LT on weekdays (5 days/week) for 1 year improved the LS stage in middle‐aged workers, which suggests that LT is especially useful for workers with LS. However, LT did not change their exercise habits.

Safe, simple, and effective multifactorial exercises involving balance and muscle strengthening are recommended for to help prevent LS. Therefore, the JOA proposed LT consisting of standing on one leg with the eyes open and performing squats.^17^ The objective of LT is mainly strengthening the muscle power of the lower extremities and improving balance, which is indispensable for ambulation and carrying out basic activities of daily living. Sakamoto et al.^18^ reported that training involving standing on one leg with the eyes open was effective to prevent falls and hip fracture among clinically defined high‐risk older people. Some studies^19^, ^20^ have reported that squats are effective to prevent functional decline and improve muscle strength in older women. Aoki et al.^7^ reported that community‐dwelling older people engaged in self‐directed home exercises. One exercise was single‐leg standing with the eyes open (three sets/day involving 1 min of standing on each leg/set), and the other was squatting or performing a chair‐rise exercise with or without hand support (three sets/day, 5–6 times/set). In their report, 3 months of training lead to improvements in seven of eight subscales on the Short Form‐8 questionnaire and various physical function tests. Ishibashi et al.^8^ also reported the effects of LT (including three sets/day of single leg standing for 1 min and three sets/day of squatting 5–10 times) on older females (mean age: 76.2 years). In their report, physical function, including gait speed, knee extension muscle power, and single leg standing time significantly increased after 2 months of training. The exercises used in this study are almost the same as those used in these previous studies, which found that LT consisting of single‐leg standing with the eyes open and performing squats were highly effective for preventing LS in older people. In this study, quadriceps muscle strength significantly increased in Company A, which suggests that LT including squatting and single‐leg standing might be effective for improving quadriceps muscle strength. Based on previous studies,^7^, ^8^, ^19^, ^20^ six repetitions of squats is not sufficient to improve muscle strength, especially in young and middle‐aged adults. However, we advised the workers in company A to perform more than six repetitions of squats until they felt muscle fatigue, which most workers were able to do. Workers who performed more repetitions of squats might have raised the average quadriceps muscle strength in company A. LT was also effective for preventing LS in young and middle‐aged workers, especially in workers with LS.

Yamada et al.^21^ reported reference values for an LS risk test according to age in Japan. In their report, the results of three kinds of LS tests gradually decreased with the age of the participants. Increasing age is one of the primary causes of decreased mobility.^22^, ^23^, ^24^ Decreased mobility has been shown to accelerate later in life, especially at age 70 years or over.^25^ Yamada et al.^23^ also indicated that increased age is not related to worse results on the three LS tests among older people with a disability. The percentage of people with a disability is much higher among older than among young and middle‐aged people. Therefore, interventions for young and middle‐aged people could be considered reasonable. LT is relatively light exercise for young and middle‐aged people. However, our results showed that LT was effective for recovering LS stage level and maintaining quadriceps muscle strength, even in young and middle‐aged adults. LT had a relatively weaker effect on workers without LS. In addition, workers without LS in Company B showed significant improvement in the stand‐up test. However, workers without LS in Company A only tended to show improvement, and workers without LS in Company B showed significantly worse LS stage and tended to show decreased quadriceps muscle strength. The improvement seen for the stand‐up test in Company B might have been the results of technical learning because workers at the follow‐up checkup already knew how to perform the stand‐up test and some workers might have trained for it. In fact, their quadriceps muscle strength tended to decrease. These results suggest that LT is effective for maintaining physical performance, such as muscle strength, even in young and middle‐aged workers without LS.

GLFS‐25 scores improved in all workers (Table 2) and workers with LS (Table 3) at company A. In all workers, activities of daily living and social functions during the last month significantly improved. Previous reports found that quadriceps strength^26^, ^27^ and balance^28^ are positively related to activities of daily living and social functions among older people. In the present study, LT, which aimed to improve lower muscle strength and balance, had positive effects on activities of daily living and social function on the GLFS‐25, even in young and middle‐aged workers. Among workers with LS, the pain element on the GLFS‐25 significantly improved. In some diseases such as knee osteoarthritis, weak muscle strength has been found to be associated with pain, and muscle training can reduce joint pain.^29^ In the present study, LT might have had a positive effect on pain in workers with LS.

Exercise habits represent another important factor in the prevention of LS. In their cohort study, Nishimura et al.^10^ reported that exercise habits during middle age affect LS in old age. Yoshimura et al.^11^ also reported that LS was less frequent in people than in those without an exercise habit. In Japan, students have to take part in physical education classes in their school. Moreover, they usually undergo a physical function test every year. However, after they start to work, they do not usually have to participate in mandatory exercise programs like physical education classes for students or physical function tests in their workplace. Most workers are not forced to participate in mandatory exercise programs and no chance to know their physical function. From this point of view, interventions for young and middle‐aged workers may be effective to help them form exercise habits and prevent the development of LS. However, the interventions in the present study did not cause workers in Companies A and B to improve their exercise habits. Information regarding their decreased physical function provided through the health checkups did not lead to improvements in their exercise habits. These results suggest that mandatory exercises might be an ideal way to help workers maintain their physical function and prevent the development of LS.

This study had several limitations. First, the number of participants was relatively small. Especially, there were far fewer female than male participants. In general, females have more orthopedic diseases and less muscle mass than males. In addition, the prevalence of LS is higher in females than in males.^4^, ^22^ Therefore, our LS test scores might be relatively higher than those in the general population. Second, this study was not population‐, but rather, occupational field‐based. The participants in our study worked at a drug or chemical company and had mostly “white‐collar” jobs, which also does not reflect the general population. Third, the health examinations were voluntary as opposed to compulsory; therefore, workers who had some pain or who worried about their motor function tended not to participate. Fourth, the number of squats performed by workers in company A was unclear.

In conclusion, the results of this longitudinal study suggest that LT is useful even for young and middle‐aged workers. Especially, LT was more effective for workers than for those without LS. However, neither checkups for LS nor a combination of checkups for LS and LT improved the workers’ exercise habits. Therefore, mandatory exercise programs at the workplace might be an ideal way to help prevent LS.

## DISCLOSURE

*Approval of the research protocol*: This study was approved by the Institutional Review Board of Mie University (approval No. U2021‐015). This study was conducted in accordance with the principles of the Declaration of Helsinki. *Informed consent*: All participants provided written informed consent prior to participating. Informed consent for the additional longitudinal study was obtained in the form of an opt‐out on the website. Those who rejected were excluded. *Registry and the registration no*. *of the study*/*trial*: N/A. *Animal studies*: N/A. *Conflict of interest*: None of the authors have any conflicts of interest to disclose.

## AUTHORS’ CONTRIBUTIONS

Akinobu Nishimura, Makoto Ohtsuki, Toshihiro Kato, Ko Kato, and Akihiro Sudo contributed to the conception and design of the study. Makoto Ohtsuki and Rie Nagao‐Nishiwaki were responsible for the participant recruitment and data collection. Akinobu Nishimura and Toru Ogura participated in the statistical analysis. Akinobu Nishimura, Makoto Ohtsuki, Toshihiro Kato, Rie Nagao‐Nishiwaki, and Yoshiyuki Senga participated in the interpretation of the data. Akinobu Nishimura drafted the manuscript. Makoto Ohtsuki, Toshihiro Kato, Rie Nagao‐Nishiwaki, Yoshiyuki Senga, Ko Kato, and Akihiro Sudo critically commented on the manuscript. Akinobu Nishimura obtained funding.

## Data Availability

The data that support the findings of this study are available on request from the corresponding author. The data are not publicly available due to privacy or ethical restrictions.
